# Seronegative Autoimmune Basal Ganglia Encephalitis Presenting as Acute Parkinsonism and Refractory Faciobrachial Seizures: A Case Report

**DOI:** 10.7759/cureus.21351

**Published:** 2022-01-17

**Authors:** Anamika Giri, Amol Andhale, Sourya Acharya, Rohan Kumar Singh, Dhruv Talwar

**Affiliations:** 1 Department of Medicine, Jawaharlal Nehru Medical College, Datta Meghe Institute of Medical Sciences (Deemed to be University), Wardha, IND; 2 Department of Radiology, Jawaharlal Nehru Medical College, Datta Meghe Institute of Medical Sciences (Deemed to be University), Wardha, IND

**Keywords:** caudate, putamen, cognitive, paraneoplastic, receptor, autoantibody

## Abstract

Autoimmune basal ganglia encephalitis (BGE) typically presents with acute onset parkinsonism and on imaging is associated with lesions in the basal ganglia. It is associated with chorea and other movement disorders. Seizures are still rare. Various autoantibodies are associated with the development of basal ganglia encephalitis. These autoantibodies are against dopamine D2 receptor (D2R) and N-methyl-D-aspartate receptor (NMDAR). Another paraneoplastic antibody known as anti-recoverin antibodies (Abs) is also associated with basal ganglia encephalitis. We report a case of a 45-year-old male who presented in this hospital with a history of cognitive dysfunction and slowness of activities for eight days and faciobrachial seizures. Magnetic resonance imaging (MRI) of the brain revealed lesions in the putamen and caudate nucleus. Infection and antibody screening were negative. The seizures were refractory to conventional antiepileptics. The patient responded to intravenous immunoglobulin (IVIG) therapy.

## Introduction

Autoimmune basal ganglia encephalitis (BGE) is characterized by the relatively rapid development of symptoms of parkinsonism such as akinesia, rigidity, and tremors. It occurs due to autoimmune inflammation of the basal ganglia structures. Various autoantibodies, namely, dopamine D2 receptor (D2R), N-methyl-D-aspartate receptor (NMDAR), and anti-LGI1, trigger this inflammation [1,2]. When basal ganglia encephalitis occurs as a paraneoplastic manifestation usually due to ovarian teratomas, then anti-recoverin antibodies (Abs) are classically associated. In these cases, there is additional development of retinopathy, also termed cancer-associated retinopathy (CAR), as these antibodies bind to specific retinal Ca2+-binding proteins [3]. The clinical presentation can also include other features such as cognitive abnormalities and seizures [4,5]. High-dose steroids, plasma exchange, intravenous immunoglobulin (IVIG), and rituximab all have been used in an attempt to treat paraneoplastic basal ganglia encephalitis, apart from targeted therapy for paraneoplastic basal ganglia encephalitis.

## Case presentation

A 45-year-old male presented to this hospital with complaints of patient abnormal irritable behavior for six days, fever with a headache for five days, recurrent abnormal movements of the face and left arm for 12 hours, and inability to speak and drowsiness for two hours. The patient was apparently alright six days back when his relatives started noticing changes in his behavior. The patient had episodes of anxiety and rage, followed by intermittent periods of depression. The patient had obsessive-compulsive behavior where he used to repeatedly rearrange his drawer and study table by taking out his books and opening them and then again rearranging them in his study table (punding). Then, the patient developed a fever that was low grade in nature and was not associated with chills and rigors associated with diffuse moderate-intensity continuous headache. There was no history of vomiting, diplopia, neck pain, drowsiness, cough, breathlessness, diarrhea, and abdominal pain.

**Figure 1:**
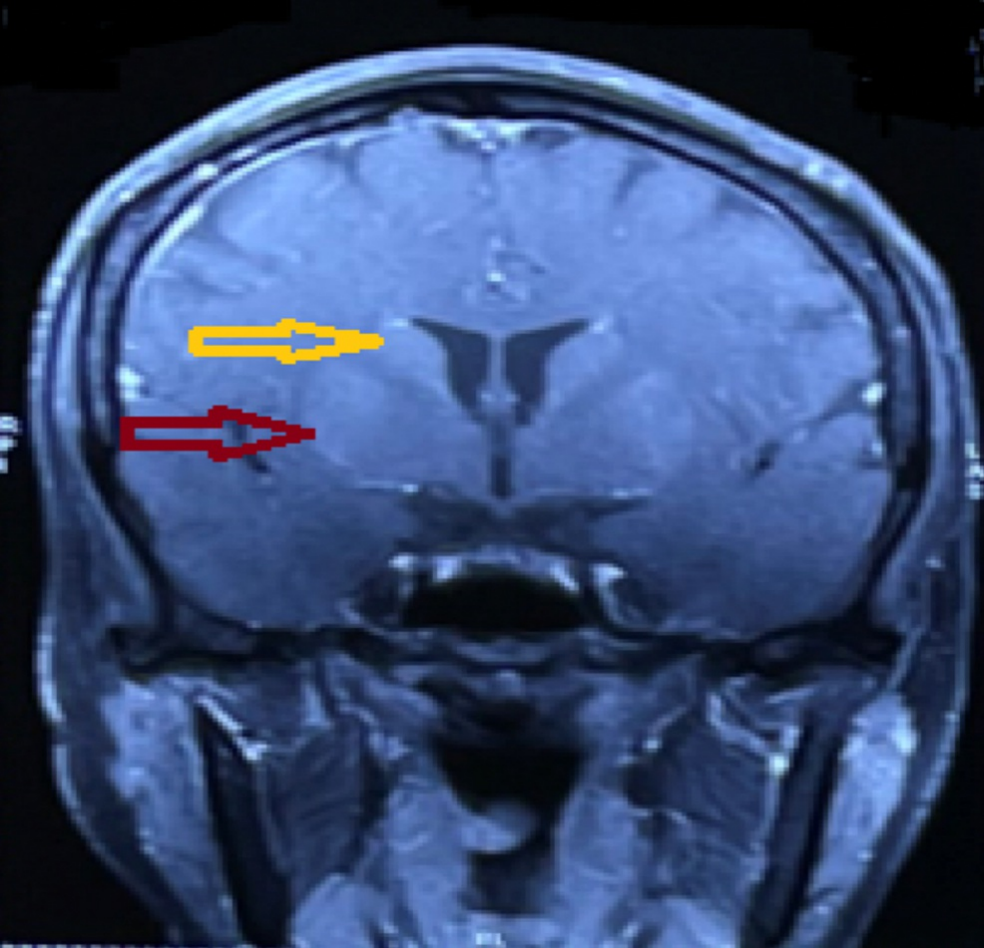
Contrast-enhanced magnetic resonance imaging coronal section of the brain showing mild enhancement of the bilateral putamen (red arrow) and bilateral caudate nucleus (yellow arrow).

**Figure 2:**
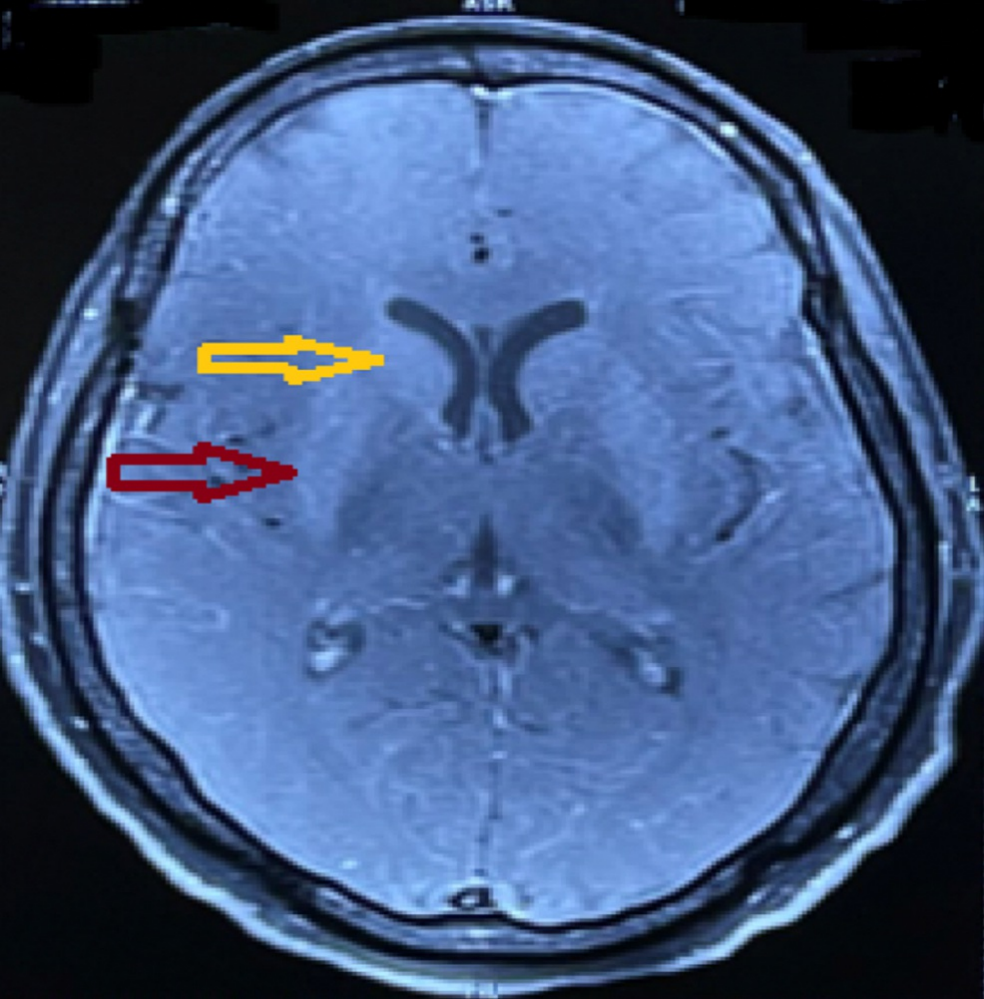
Contrast-enhanced magnetic resonance imaging axial section of the brain showing mild enhancement of the bilateral putamen (red arrow) and bilateral caudate nucleus (yellow arrow) with no abnormal enhancement of the meninges.

**Figure 3:**
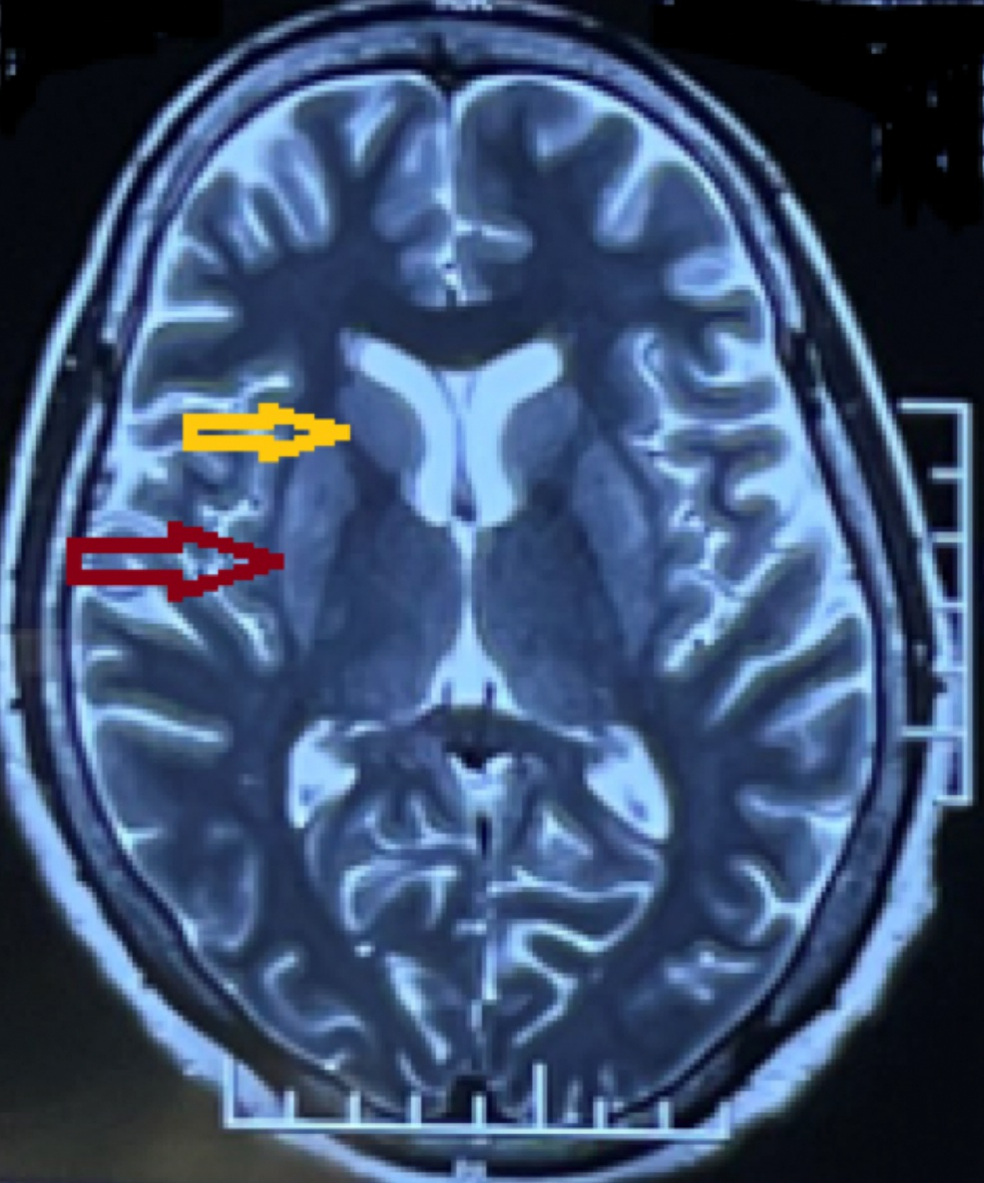
T2-weighted magnetic resonance imaging axial section of the brain showing increased signal intensity in the bilateral putamen (red arrow) and bilateral caudate nucleus (yellow arrow).

**Figure 4:**
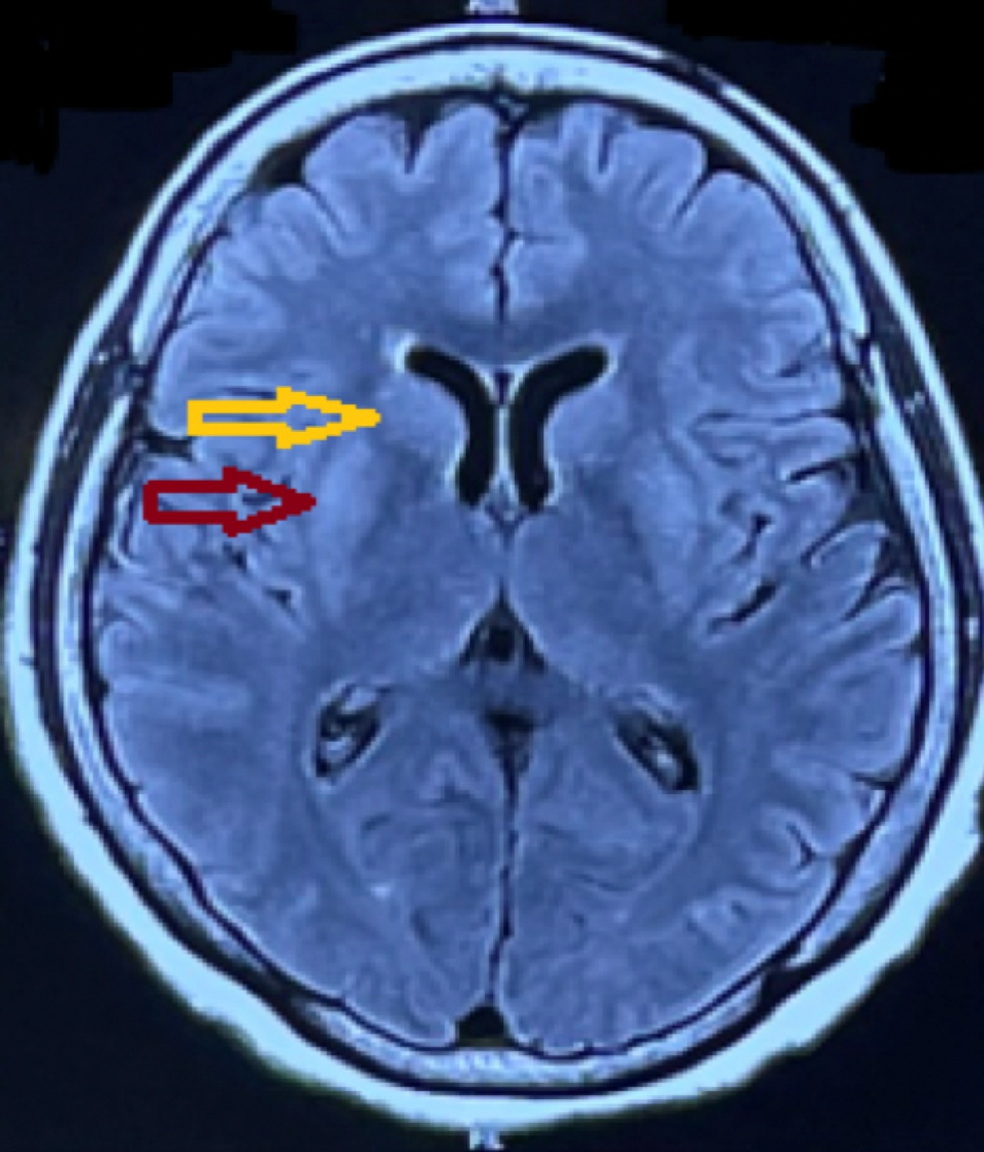
FLAIR magnetic resonance imaging axial section of the brain showing increased signal intensity in the bilateral putamen (red arrow) and bilateral caudate nucleus (yellow arrow).

The patient had the first episode of abnormal contractions of the left face and left hand, which lasted roughly for about five minutes and was not associated with any loss of consciousness but was associated with difficulty in speaking. The patient had a history of similar two episodes on the day of admission, after which there was decreased consciousness and inability to speak. Then, the patient was immediately brought to this hospital by relatives. The patient had an episode of seizure in the casualty, consisting of an abrupt onset of abnormal contractions of the left side of the face and left hand. Lorazepam 2 mg injection was given intravenously, and the seizure subsided. His GCS score was 8 (E2, M4, and V2), for which an urgent magnetic resonance imaging (MRI) of the brain was advised, and the patient was intubated and shifted to intensive care unit (ICU). Plain and contrast brain MRI was done, which was suggestive of bilateral enhancement of putamen and caudate nucleus with no changes in the meninges. Axial section diffusion-weighted images showed diffusion restriction in bilateral putamen and caudate nucleus. T2-weighted images and FLAIR images were suggestive of increased signal intensity in bilateral putamen and caudate nucleus (Figures 1-4).

Examination in ICU revealed rigidity in all four limbs with cogwheeling at bilateral wrist joints. The patient continued to have recurrent episodes of seizures (Video 1) for which antiepileptics such as phenytoin, lamotrigine, and levetiracetam were initiated, but the patient did not respond to it. The patient was started prophylactically on acyclovir injection. Intermittent seizures continued, for which thiopental was started intravenously.

**Video 1:** Recurrent episodes of seizure in the present case.

All routine blood examinations (complete blood count, renal function test, and liver function test) revealed no obvious abnormality. Serum procalcitonin was normal. Malaria antigen test, IgM scrub typhus, and IgM dengue were negative. Chest X-ray, abdominal USG, and chest and abdominal CT were normal. ECG and bedside 2D echo were normal. The patient’s CSF examination was done, which revealed the following: total cell count, 15 cells, all lymphocytes; CSF sugar, 98 mg/dL; and protein, 98 mg/dL. CSF culture and herpes simplex virus (HSV) PCR were negative, ruling out infectious encephalitis.

Autoantibodies in serum, as well as CSF for NMDAR, LGI1, anti-recoverin, anti-contactin-associated protein 2 (CASPR2), and anti-VGKC, were all negative. Based on the serological findings, MRI report, and clinical findings, a diagnosis of seronegative basal ganglia encephalitis was made. The patient was started on intravenous methylprednisolone 1 g daily for three days and intravenous immune globulin 400 mg/kg/day for five days, and acyclovir injection was stopped. The patient showed dramatic improvement over a course of a week and was eventually discharged on oral steroids.

## Discussion

Autoimmune processes that cause a variety of neuropsychiatric symptoms are becoming more widely recognized, resulting in a radical transformation in our knowledge of autoimmune encephalitis (AE). They are an autoantibody-mediated encephalitis group that responds effectively to immunotherapy. They manifest a wide range of clinical symptoms, including cognitive impairment, behavioral alterations, personality changes, seizures, aberrant movements, autonomic disturbances, and sensorium loss. Initial diagnosis criteria were heavily reliant on antibody testing and treatment response [6]. However, because antibodies may not always be detected, these criteria may not always be feasible. Seronegative (or antibody-negative) autoimmune encephalitis (SAE) with probable immunological origin based on clinical and imaging characteristics is a diagnostic difficulty when there are no detectable autoantibodies in serum or CSF.

One of the least recognized types of autoimmune encephalitis is basal ganglia encephalitis. Patients with autoimmune basal ganglia encephalitis have neurological signs of parkinsonism, such as involuntary movements, stiffness, and tremors, which are associated with basal ganglia lesions. Autoantibodies to the dopamine D2 receptor (D2R) and the N-methyl-D-aspartate receptor (NMDAR) are usually linked to autoimmune basal ganglia encephalitis. It is also recognized that anti-recoverin antibodies (Abs) are paraneoplastic antibodies that attach to certain retinal Ca2+-binding proteins, thus causing retinopathy. It is not always necessary that antibodies will be detected in a case of basal ganglia encephalitis. It is a diagnosis made on neuroimaging [7]. Patients with autoimmune basal ganglia encephalitis who demonstrated basal ganglia atrophy or gliosis on acute scans experienced chronic cognitive and mental impairment, according to previous studies [8]. Treatment for suspected autoimmune encephalitis is frequently provided empirically. Steroids and/or IVIG may be used. Initial therapies for a cell-surface or synaptic antibody disease may involve IVIG, plasmapheresis, and/or steroids.

## Conclusions

Autoimmune BGE is a distinct entity characterized by the development of various neuropsychiatric manifestations; acute parkinsonian features such as akinesia, rigidity, and tremors; and neuroimaging results revealing lesions in basal ganglia nuclei. Several autoantibodies are associated with BGE, including paraneoplastic antibodies. Any patient with features of BGE should be screened for malignancy. Treatment is usually with steroids, IVIG, rituximab, and plasmapheresis.
